# Effects of personal growth initiative on occupational engagement of college students in the uncertain social context: A cross-lagged model and a moderated mediation model

**DOI:** 10.3389/fpsyg.2022.988737

**Published:** 2022-09-21

**Authors:** Zhun Gong, Xinian Jiao, Meiqian Zhang, Qunzhen Qu, Baicai Sun

**Affiliations:** ^1^Normal College, Qingdao University, Qingdao, China; ^2^School of Economics and Management, Shanghai Maritime University, Shanghai, China

**Keywords:** occupational engagement, personal growth initiative, vocational identity, big five personality traits, longitudinal study

## Abstract

In recent years, the international social context has become increasingly volatile, uncertain, complex, ambiguous (VUCA), and college students need a high level of long-term occupational engagement to cope with the unpredictability of the current employment environment. In this context, this study used a longitudinal design to explore the relationship between personal growth initiative and occupational engagement among college students and, based on this, further explored the role of vocational identity and Big Five personality traits in it. This study used a questionnaire survey method and the study participants were 700 college students in Shandong, China. And the time interval between the two questionnaire measurements was 4 months, with 559 final valid participants (182 males and 377 females). The following results were found in this study: (1) The cross-lagged model found that personal growth initiative was a significant positive predictor of occupational engagement. (2) The moderated mediation model found that vocational identity mediated the relationship between personal growth initiative and occupational engagement, and that neuroticism among the Big Five personality traits played a moderating role, i.e., individuals with higher level of neuroticism personality trait had a relatively weaker positive predictive effect of vocational identity on occupational engagement. This study concludes that colleges and universities need to understand students’ interests and personality traits and provide more targeted career education (e.g., intentional growth training and cognitive behavioral therapy) to promote their personal growth initiatives, reduce their neuroticism levels and in turn enhance their vocational identity in order to help college students achieve long-term sustainable occupational engagement in the uncertain social context.

## Introduction

In recent years, the international social context has become increasingly volatile, uncertain, complex, ambiguous (VUCA) which has led to the current employment environment becoming unpredictable and variability (Noda, 2020). Due to the uncertainty and dynamic changes of the current employment environment, individual career paths are no longer linear or predictable, and career researchers have recognized this trend and emphasized the importance of actual individual behaviors in career development (Kim et al., 2018). And occupational engagement is an essential behavior that leads to successful and satisfying career development for college students (Cox et al., 2015). Occupational engagement refers to an individual’s efforts to research and engage in career-related activities and is a habitual behavior rather than a specific behavior to make career decisions (Krieshok et al., 2009). It emphasizes the importance of individuals engaging in relevant occupational activities and reflecting on these experiences, and that individuals learn and develop new occupational skills through occupational engagement, as an experiential learning process. Park and Yoo (2020) found that college students with higher levels of occupational engagement tended to have higher levels of career adaptability in an uncertain employment environment. And college students increase their career skills and knowledge reserves in the process of occupational engagement, which helps them to cope with the difficulties they encounter in their career development (Kim et al., 2021). Therefore, how to improve the occupational engagement of college students has become an urgent issue to be solved in the current uncertain social context.

### Personal growth initiative and occupational engagement

Personal growth initiative is an individual’s tendency to actively and consciously refine the skills needed in the growth process (Robitschek, 1998). Self-determination theory emphasizes that an autonomous motivational orientation is often associated with higher levels of mental health and high performance in individuals (Deci and Ryan, 2008; Deci et al., 2017). Personal growth initiative emphasizes the dynamic process of individual self-directed change, and research has found that employees with higher levels of personal growth initiative show greater perseverance in finding opportunities for self-growth (Yakunina et al., 2013). Career construction theory (Savickas, 2002, 2013) suggests that individuals continually adjust and adapt to career development based on acquired experiences, which is a dynamic developmental process. Sharma and Rani (2013) found that college students with high personal growth initiative are open to challenges in their career development and always strive for excellence. Personal growth initiative can enable behavioral changes in an individual’s career development and help individuals sustain their occupational engagement, therefore, personal growth initiative can be considered as a launching point for college students to make occupational engagement (Ryan and Deci, 2000; Deci and Ryan, 2008). And Vuyk et al. (2020) conducted a cross-cultural analysis and found that personal growth initiative was moderately positively correlated with occupational engagement, and the relation between personal growth initiative and occupational engagement was stronger among Chinese college students compared to Paraguayan college students. It indicates that in the Chinese context, personal growth initiative is an important factor influencing occupational engagement. Furthermore, upon entering the workplace, individuals with high personal growth initiative are immersed in their work longer, are more engaged, and are focused on their responsibilities, because they connect work to emotions and proactively seek and take advantage of development opportunities as they explore their careers (Srivastava and Bajpai, 2021). This study, based on career construct theory, argues that college students’ personal growth initiative will promote occupational engagement, which may have long-term benefits for individual career development.

### The mediating role of vocational identity

Vocational identity refers to an individual’s clear and stable picture of his or her goals, interests and talents (Holland et al., 1980; Zhang et al., 2018). Role identity theory suggests that individuals derive their sense of self and meaning from the different social roles they play in society (e.g., friend, partner, student, or manager) and emphasizes that an individual’s self-view shapes his or her identity in a particular social role (Stryker and Burke, 2000). Vocational identity is the self-perception that individuals hold of their abilities to match the requirements of the job, and personal growth initiative happens to be a positive perspective, which has been found to be closely related to vocational identity, as positive self-perceptions lead to the development of a stronger identity associated with the role (Robitschek and Cook, 1999; Deng et al., 2018; Jian and Chen, 2021). Thus, personal growth initiatives may help to promote the development of individual vocational identity. Role identity theory also emphasizes that individuals will develop self-concept apprehension and form an identity with their roles according to the different requirements of the roles they play in society, which will guide their behaviors (Farmer et al., 2003); therefore, when individuals have a strong sense of vocational identity, it will facilitate the achievement of self-directed and value-driven career goals in career development and help to increase individuals’ occupational engagement (Hall and Cheston, 2002; Vuyk et al., 2020). It has been found that vocational identity is an important indirect factor influencing individual career development, and research has shown that vocational identity mediates the relationship between perceived overqualification and career planning, i.e., individuals who perceive overqualification tend to develop higher levels of vocational identity and engage in more explicit career planning (Ma et al., 2020).

### The moderating effects of the big five personality traits

Personality is a system of interconnected, interdependent traits that together drive behavior (Dai et al., 2004). Goldberg (1990) proposed the “Big Five” or Five-Factor Model (FFM) of personality on the basis of a large number of previous studies, and five factors included Neuroticism(N), Extraversion(E), Openness(O), Agreeableness(A), Conscientiousness(C). After a lot of research, the “big five” model has been accepted by many personality psychologists. Personality traits can have an impact on an individual’s career development, trait activation theory suggests that personality traits are individual responses to trait-related contextual cues (Tett and Guterman, 2000), and that individual personality traits are associated with the knowledge, skills, and abilities they possess, and that individual personality traits influence the acquisition of knowledge, the development of skills, and the enhancement of abilities, and further influences individual behavior (Liu et al., 2020). Studies have found that individuals with higher levels of openness are more open to new experiences and positively influence their own occupational engagement, while individuals with higher levels of neuroticism negatively influence their own occupational engagement (Janssens et al., 2019; Srivastava and Bajpai, 2021). It has been suggested that personality traits are also highly associated with career adaptability, which is closely related to future career development, and that different personality traits have different effects on career adaptability. Specifically, openness, agreeableness, conscientiousness, and extroversion personality traits positively influence youth’s career adaptability, while neuroticism negatively influences youth’s career adaptability (Bacanli and Sarsıkoğlu, 2021). Vocational identity involves an individual’s self-perception and self-awareness and, therefore, may interact with an individual’s personality traits to influence his or her occupational engagement.

### Research objectives and hypotheses

In recent years, the current employment environment become unpredictable and variability (Noda, 2020), and individual career paths are no longer linear or predictable (Kim et al., 2018), thus career researchers emphasized the importance of occupational engagement in career development. Therefore, how to improve the occupational engagement of college students has become an urgent issue to be solved in the current uncertain social context. From a theoretical perspective, this study is an application and extension of career construction theory in the uncertain social context. Career construction theory (Savickas, 2002, 2013) suggests that individuals continually adjust and adapt to career development based on acquired experiences, which is a dynamic developmental process. Based on this, this study attempts to explore the impact of personal growth initiative on occupational engagement among college students through a longitudinal design. And, this study further explores the mechanisms and boundary conditions of personal growth initiative on occupational engagement based on role identity theory (Farmer et al., 2003) and trait activation theory (Tett and Guterman, 2000; Liu et al., 2020). From a practical perspective, this study provides suggestions on how to help college students improve their occupational engagement in order to achieve more satisfying career development in an uncertain social context. The objectives and hypotheses of this study are as follows.

(1) To what extent does personal growth initiative predict occupational engagement among Chinese students? Based on career construction theory (Savickas, 2002, 2013), this study proposed the following hypothesis and tested by constructing a cross-lagged model through a longitudinal design.

*H1: College students’ personal growth initiative can positively predict their occupational engagement*.

(2) What are the mechanisms and boundary conditions of the influence of personal growth initiative on occupational engagement? Based on role identity theory (Farmer et al., 2003) and trait activation theory (Tett and Guterman, 2000; Liu et al., 2020), this study proposed the following hypotheses and tested by constructing a moderated mediation model.

*H2: Moderated by Big Five personality traits, college students’ vocational identity plays a mediating role in the relationship between personal growth initiative and occupational engagement*.

*H2a: College students’ vocational identity mediates the relationship between personal growth initiative and occupational engagement*.

*H2b: College students’ Big Five personality traits (including neuroticism, extraversion, openness, agreeableness, conscientiousness) play a moderating role between vocational identity and occupational engagement*.

## Materials and methods

### Participants

The participants were university students in Shandong Province, China, who were followed up with two questionnaires, 4 months apart. The first was in September 2021 (T1), with 700 questionnaires returned, and the second was in January 2022 (T2), with 668 questionnaires returned. After eliminating invalid questionnaires according to the polygraph questions, we finally got 559 valid questionnaires, among which, 182 were male and 377 were female; 203 were freshmen, 188 were sophomores, 82 were juniors and 86 were seniors.

### Measurement tools

#### Personal growth initiative

This study used the Personal Growth Initiative Scale-II (PGIS-II) developed by Robitschek et al. (2012). The scale is a 16-item scale consisting of 4 subscales: Readiness for Change, Planfulness, Using Resources, and Intentional Behavior. For example, “I can tell when I am ready to make specific changes in myself” and “I set realistic goals for what I want to change about myself.” The 6-point response scale had endpoints ranging from 0 (Disagree strongly) to 5 (Agree strongly). The higher the total score, the higher the individual’s personal growth initiative. In the present study, the Cronbach’s alpha coefficient for the scale was 0.90 for T1 and 0.89 for T2. Therefore, the scale had good reliability.

#### Vocational identity

This study used the Vocational Identity Status Assessment (VISA) revised by Zhang et al. (2018) in the Chinese context, which has a total of 30 items. The scale consists of 3 dimensions: Career Exploration, Career Commitment, and Career Reconsideration. For example, “No other career is as appealing to me as the one I expect to enter” and “Becoming a worker in my chosen career will allow me to become the person I dream to be.” Using a 5-point Likert scale ranging from 1 (Strongly disagree) to 5 (Strongly agree), the higher the total score, the higher the individual’s sense of vocational identity. In the present study, the Cronbach’s alpha coefficient for the scale was 0.86 for T1 and 0.83 for T2. Therefore, the scale had good reliability.

#### Occupational engagement

The Occupational Engagement Scale-Student version (OES-S) developed by Cox et al. (2015) was used in this study. The scale has a total of nine items and is a unidimensional scale. Response options ranged from 1 (Unlike me) to 5 (Like me). For example, “I talk about my career choices with family or friends” and “I visit places I’m interested in working so I can learn more about them.” The higher the total score, the higher the individual’s occupational engagement. In the present study, the Cronbach’s alpha coefficient for the scale was 0.83 for T1 and 0.70 for T2. Therefore, the scale had good reliability.

#### Big five personality traits

The Chinese version of the NEO Personality Inventory (NEO-PI-R) revised by Dai et al. (2004) was used. The scale has 30 items and consists of five dimensions: Neuroticism(N), Extraversion(E), Openness(O), Agreeableness(A), Conscientiousness(C). For example, “I really enjoy talking to people” and “When I’m under a lot of pressure, sometimes I feel like I’m being torn to pieces.” The scale used a 5-point Likert scale, ranging from 1 (Disagree strongly) to 5 (Agree strongly). In the present study, the Cronbach’s alpha coefficient for the scale was 0.75. Therefore, the scale had good reliability.

### Data analysis

This study used SPSS 26.0 for reliability analysis, descriptive statistics and correlation analysis referring to Shi et al. (2021). And to test for common method bias, this study referring to Podsakoff et al. (2003) performed Harman’s one-way test using SPSS 26.0. To examine the relationship between personal growth initiative and occupational engagement among college students, this study constructed a cross-lagged model using Mplus 8.3 referring to Kobayashi et al. (2020). Furthermore, to examine the mechanisms and boundary conditions of the influence of personal growth initiative on occupational engagement, this study constructed a moderated mediation model using Mplus 8.3 and referred to the data collection strategy for the moderated mediation model provided by Xu and Lv (2018), i.e., the independent and moderating variables use data collected at T1 and the mediating and dependent variables use data collected at T2.

## Result

### Common method bias test

In this study, referring to Podsakoff et al. (2003), the common method bias test was performed using Harman’s one-way test, and the variance explained without rotation by the first factor was 13.15% for T1 and 19.80% for T2, which were less than 40%, indicating that there was no common method bias in this study.

### Descriptive statistics and correlation analysis

The results of descriptive statistics and correlation analysis are shown in Table 1.

**TABLE 1:** Descriptive statistics and correlation analysis.

	1	2	3	4	5	6	7	8	9	10	11	12	13
1. T1 personal growth initiative	1												
2. T1 vocational identity	0.64**	1											
3. T1 occupational engagement	0.55**	0.58**	1										
4. T1 neuroticism	0.02	0.12**	0.003	1									
5. T1 conscientiousness	0.47**	0.30**	0.30**	0.01	1								
6. T1 agreeableness	−0.03	0.16*	0.03	0.32**	−0.15**	1							
7. T1 openness	0.22**	0.27**	0.18**	0.21**	0.20**	0.14**	1						
8. T1 extraversion	0.24**	0.22**	0.21**	0.12**	0.27**	0.17**	0.39**	1					
9. T2 personal growth initiative	0.77**	0.50**	0.41**	−0.01	0.31	0.05	0.12**	0.18**	1				
10. T2 vocational Identity	0.73**	0.66**	0.46**	0.01	0.26**	0.02	0.18**	0.13**	0.59**	1			
11. T2 occupational engagement	0.67**	0.57**	0.67**	−0.13**	0.27**	−0.09**	0.13**	0.14**	0.51**	0.70**	1		
12. Gender	0.12**	−0.02	0.03	0.003	0.16**	−0.10*	0.02	0.02	0.05	0.07	0.06	1	
13. Grade level	0.18**	0.28**	0.20**	0.002	0.11**	0.07	0.02	0.02	0.15**	0.15**	0.16**	−0.18**	1
*M* (*SD*)	3.69 (0.55)	3.51 (0.40)	3.46 (0.67)	3.22 (0.48)	3.22 (0.44)	3.29 (0.56)	3.08 (0.41)	2.95 (0.51)	3.72 (0.54)	3.60 (0.39)	3.66 (0.50)		

*p < 0.05 and **p < 0.01.

### A cross-lagged model test of personal growth initiative and occupational engagement

**FIGURE 1:**
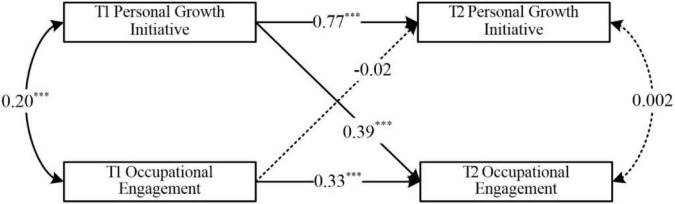
A cross-lagged model of personal growth initiative and occupational engagement *p < 0.05, **p < 0.01, and ***p < 0.001.

To examine the relationship between personal growth initiative and occupational engagement among college students, this study constructed a cross-lagged model referring to Kobayashi et al. (2020). Gender and grade were used as control variables, and data were analyzed using Mplus 8.3, and the results are shown in Figure 1: T1 personal growth initiative can significantly and positively predict T2 occupational engagement (β = 0.39, *p* < 0.001) and T2 personal growth initiative (β = 0.77, *p* < 0.001). T1 occupational engagement can significantly and positively predict T2 occupational engagement (β = 0.33, *p* < 0.001), but not T2 personal growth initiative (β = −0.02, *p* = 0.47). And T1 personal growth initiative is significantly and positively correlated with T1 occupational engagement (*r* = 0.20, *p* < 0.001), while T2 personal growth initiative is not significantly correlated with T2 occupational engagement (*r* = 0.002, *p* > 0.05). The results of this study suggest that college students’ personal growth initiative is an antecedent variable of occupational engagement. Therefore, hypothesis 1 is accepted, i.e., college students’ personal growth initiative can significantly and positively predict occupational engagement.

### A moderated mediation model test

This study used Mplus 8.3 to further examine the mechanisms and boundary conditions of the influence of personal growth initiative on occupational engagement referring to Xu and Lv (2018), with T1 personal growth initiative as the independent variable, T2 occupational engagement as the dependent variable, T2 vocational identity as the mediating variable, T1 Big Five personality traits (including conscientiousness, agreeableness, openness, extraversion, and neuroticism) as moderating variables, and gender and grade as control variables, the moderated mediation model was constructed.

The results are shown in Table 2, T1 personal growth initiative significantly and positively predicts T2 vocational identity (β = 0.73, *p* < 0.001, 95% CI [0.68, 0.77]) and T2 vocational identity significantly predicts T2 occupational engagement (β = 0.45, *p* < 0.001, 95% CI [0.38, 0.53]). Therefore, hypothesis 2a is accepted, i.e., vocational identity mediates the relationship between personal growth initiative and occupational engagement.

**TABLE 2:** Path analysis of the moderated mediation model.

	Predictors	Model fit indices		Path coefficients				
		*F*	*R* ^2^	β	*t*	*p*	95% CI	
T2 vocational identity	Gender	158.07***	0.54	−0.05	−2.09*	0.04	−0.10	−0.003
	Grade			0.003	0.27	0.79	−0.02	0.02
	T1 personal growth initiative			0.73	31.01***	<0.001	0.68	0.77
T2 occupational engagement	Gender	46.07***	0.57	−0.02	−0.73	0.46	−0.08	0.04
	Grade			0.02	1.39	0.16	−0.01	0.05
	T1 personal growth initiative			0.32	6.11***	<0.001	0.22	0.40
	T2 vocational identity			0.45	9.90***	<0.001	0.38	0.53
	T1 conscientiousness			−0.03	−0.71	0.48	−0.10	0.04
	T1 agreeableness			−0.06	−1.75	0.08	−0.12	−0.002
	T1 openness			0.05	1.36	0.17	−0.01	0.11
	T1 extraversion			0.03	0.95	0.35	−0.02	0.90
	T1 neuroticism			−0.13	−4.16***	<0.001	−0.18	−0.08
	T2 vocational identity × T1 conscientiousness			−0.05	−1.32	0.19	−0.11	0.01
	T2 vocational identity × T1 agreeableness			−0.01	−0.35	0.73	−0.07	0.05
	T2 vocational identity × T1 openness			0.001	0.02	0.98	−0.05	0.06
	T2 vocational identity × T1 extraversion			0.06	1.93	0.06	0.00	0.11
	T2 vocational identity × T1 neuroticism			−0.08	−2.29*	0.02	−0.13	−0.03

*p < 0.05, **p < 0.01, and ***p < 0.001.

The interaction term between T1 neuroticism and T2 vocational identity significantly and negatively predicts T2 occupational engagement (β = −0.08, *p* = 0.02, 95% CI [−0.13, −0.03]). Hence, hypothesis 2b is partly accepted, i.e., neuroticism plays a moderating role between vocational identity and occupational engagement, but the moderating effects of agreeableness, conscientiousness, openness, and extraversion are not significant.

**FIGURE 2:**
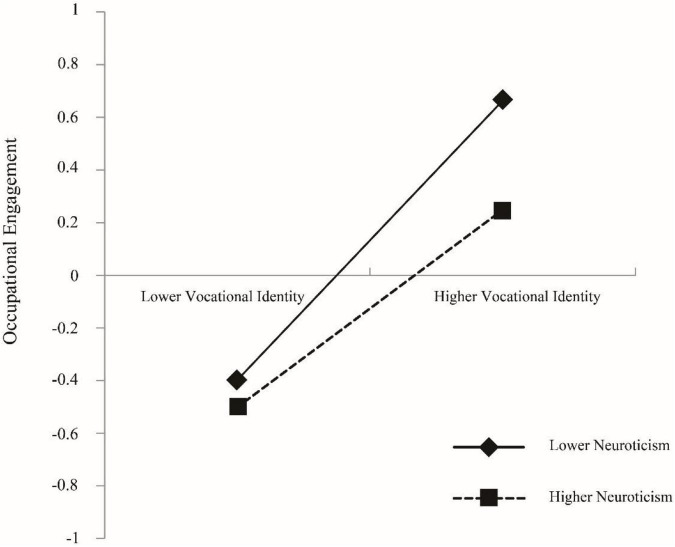
The moderating effect of neuroticism between vocational identity and occupational engagement.

To further examine the moderating effect of neuroticism personality trait between vocational identity and occupational engagement, this study referred to Aiken and West (1991) to conduct a simple slope test and plotted the moderating effect using the mean plus or minus standard deviation ($\bar{\text{X}}\pm \text{S}$) of neuroticism personality trait as the basis for higher and lower grouping, and the results are shown in Figure 2. College students with higher levels of neuroticism personality trait have a relatively lower positive relationship between vocational identity and occupational engagement (β = 0.37, *p* < 0.001), while those with lower levels of neuroticism personality trait have a higher positive relationship between vocational identity and occupational engagement (β = 0.53, *p* < 0.001).

## Discussion

This study examined the relationship between personal growth initiative and occupational engagement of college students in the VUCA environment using a longitudinal design. By constructing a cross-lagged model, we found that personal growth initiative significantly and positively predicted occupational engagement. Based on this, a moderated mediation model was further constructed to verify that vocational identity mediated the relationship between personal growth initiative and occupational engagement, and that neuroticism personality traits moderated the relationship between vocational identity and occupational engagement, i.e., individuals with higher levels of neuroticism personality traits had a relatively weaker positive predictive effect of vocational identity on occupational engagement.

### Positive predictive effect of college students’ personal growth initiative on occupational engagement

This study finds a significant positive correlation between personal growth initiative and college students’ occupational engagement, which is in line with previous studies (Sharma and Rani, 2013; Vuyk et al., 2020). And the results of the cross-lagged model show that T1 personal growth initiative can significantly and positively predict T2 college students’ occupational engagement. The possible reason for this is that individuals with high personal growth initiative are willing to spend more time on occupational engagement (Sharma and Rani, 2013). Therefore, enhancing the personal growth initiative of college students can help promote the level of individual occupational engagement, which is beneficial to individual career development. The results of this study further support the view of self-determination theory and career construct theory that individuals with high autonomy motivation usually achieve more positive outcomes in career development (Savickas, 2013; Deci et al., 2017). In the current VUCA environment, the uncertainty and ambiguity of the social context make individuals face certain challenges when it comes to career development, such as employment difficulties and job instability. Individuals with a higher level of personal growth initiative will be more active in seeking opportunities and resources in their career development and reserve career skills to increase their occupational engagement so that they can be successfully employed and increase their job stability, therefore, the personal growth initiative of college students is beneficial to their career development.

### The mediating role of vocational identity

This study finds that vocational identity mediates the relationship between personal growth initiative and occupational engagement. The possible reason for this is that individuals with higher personal growth initiative tend to acquire career-related information and make positive self-change to further integrate career information with the self as a way to promote their vocational identity (Weigold et al., 2020; Kim et al., 2021). Moreover, individuals with higher levels of vocational identity have a better understanding of their career development needs (Diemer and Blustein, 2007), and are consequently able to make more appropriate and extensive occupational engagement. Therefore, increasing the personal growth initiative of college students is beneficial to enhance their vocational identity, which in turn helps them achieve a more appropriate and extensive occupational engagement. This will facilitate college students’ career preparation and help them to make precise management on the path of career construction.

### The moderating effect of neuroticism

Neuroticism plays a moderating role between vocational identity and occupational engagement, specifically, individuals with higher levels of neuroticism have a weaker positive predictive effect of vocational identity on occupational engagement. The results of this study support the view of trait activation theory that neurotic trait personality traits are activated in the scenario of career development planning and management by college students, and that the influence of vocational identity on occupational engagement changes under the effect of neurotic personality traits. This may be due to the fact that individuals with higher levels of neuroticism tend to experience more negative emotions (Costa and McCrae, 1992; Djurkovic et al., 2006), including anger, anxiety, depression, insecurity, helplessness, low self-esteem, mood swings, etc., but their lack of ability to effectively deal with negative emotions, as well as less use of effective or self-appropriate stressful events at work (Zhong and Ling, 2014), thus leading to the emergence of a large number of ruminative behaviors and strong concerns about future career development (Muris and Ollendick, 2005; Barnhofer and Chittka, 2010; Merino et al., 2016). Thus, college students with high levels of neuroticism focus more on the unfavorable aspects of their careers and may develop withdrawal, which hinders occupational engagement.

### Theoretical implications

First, this study is an application of career construction theory (Savickas, 2002, 2013) in the current uncertainty social context, where stable and secure traditional employment relationships are being disrupted by short-term, highly flexible employment practices (Guan and Li, 2015), and where individual career development is complicated by the volatility of the employment environment (Noda, 2020). Second, career construction theory emphasizes the dynamic developmental process in which individuals continuously adjust and adapt to their careers based on the experiences they acquire (Rudolph et al., 2019). While previous studies on personal growth initiative and occupational engagement have been limited to cross-sectional studies (Sharma and Rani, 2013; Vuyk et al., 2020), this study attempted to construct a cross-lagged model using a longitudinal design and found a positive predictive effect of T1 personal growth initiative on T2 occupational engagement. It indicates that there may be a long-term benefit of personal growth initiative on occupational engagement. Third, based on role identity theory (Farmer et al., 2003) and trait activation theory (Tett and Guterman, 2000), this study further explored the mechanism and boundary condition of college students’ personal growth initiative for occupational engagement, which extended career construction theory. The mediating role of vocational identity implies a role identity mechanism in explaining the effect of personal growth initiative on occupational engagement. This suggests that college students with higher personal growth initiative are more likely to generate higher levels of vocational identity, which in turn promotes higher levels of occupational engagement. Furthermore, trait activation theory emphasizes a unique perspective from interaction psychology that explores the interaction between external contexts and intrinsic traits of individuals, and the predictive role of this interaction on individual behavior (Zagenczyk et al., 2017; Liu et al., 2020). Based on this, this study found that neuroticism is an important boundary condition for the mediating relationship between personal growth initiative and occupational engagement through vocational identity. This suggests that career-related role identification processes may be influenced by the level of individual neuroticism personality trait, specifically, individuals with higher levels of neuroticism have a weaker positive predictive effect of vocational identity on occupational engagement.

### Practical implications

The employment environment has become unpredictable and variability due to the fact that it is currently in an uncertain social context, that is, the international social environment is becoming increasingly volatile, uncertain, complex, ambiguous (VUCA) (Noda, 2020). Therefore, individuals’ career paths are no longer linear or predictable, and career researchers have recognized this trend and emphasized the importance of actual individual behavior in career development (Kim et al., 2018). Research has found that occupational engagement is an essential behavior that leads to successful and satisfying career development for college students (Cox et al., 2015), and researchers are beginning to look at ways to improve the level of occupational engagement of college students. In this context, this study finds that college students’ personal growth initiative positively predicts vocational identity and occupational engagement, therefore, colleges may consider intervening in students’ personal growth initiative to help them improve their vocational identity and occupational engagement. Thoen and Robitschek (2013) have developed an intervention that can effectively increase the personal growth initiative of college students, namely, intentional growth training (IGT). It contains understanding the process of change (through educational lecture), gaining insight into current life circumstances and emotional states (through thinking about a past attempt to change oneself), exploring issues of stress and risk taking (through describing that change usually involves stepping outside of one’s comfort zone), and setting goals and developing action plans for the future (through the planning of a personal growth activity to complete within 1 week). Based on this, colleges may consider offering IGT-related course practices to promote students’ personal growth initiatives, further improve their vocational identity and occupational engagement, and help them develop well in the current uncertain social context. Furthermore, college students with higher levels of neuroticism have a weaker positive predictive effect of vocational identity on occupational engagement. It indicates that higher levels of neuroticism are detrimental to the improvement of occupational engagement among college students. Therefore, career planning education in college should pay more attention to students’ personality traits, help students with higher levels of neuroticism learn to deal with the negative emotions that may arise in their career development, and encourage them to try to improve their level of occupational engagement. At the same time, cognitive behavioral therapy (CBT) can be effective in reducing the level of neuroticism among college students (Hedman et al., 2014). Therefore, colleges may consider offering elective courses in cognitive behavioral therapy and encourage students who need it to participate in the relevant courses.

### Research limitations and future directions

First, this study was conducted in a Chinese context, but there may be cultural differences in the mechanisms and boundary conditions for the role of personal growth initiative on occupational engagement, so follow-up studies may attempt to conduct cross-cultural research to test the cross-cultural consistency of the findings. Second, the participants in this study were all college students, and subsequent studies could expand the group span, such as further investigating groups of doctoral students, high school students, or vocational and technical school students, which would help to examine the development of occupational engagement in groups with different educational backgrounds. Third, this study adopted a questionnaire survey method to explore the mechanisms and boundary conditions of the role of personal growth initiative on occupational engagement. It provides a theoretical basis for subsequent studies, which can build on this foundation and carry out intervention practices to improve college students’ vocational identity and occupational engagement through interventions on personal growth initiative (e.g., IGT) and neuroticism (e.g., CBT).

## Conclusion

In summary, personal growth initiative of college students can enhance their vocational identity, which in turn increases their occupational engagement, and neuroticism personality trait also interacts with vocational identity in occupational engagement. This study concludes that colleges and universities need to understand students’ interests and personality traits and provide more targeted career education (e.g., intentional growth training and cognitive behavioral therapy) to promote their personal growth initiatives, reduce their neuroticism levels and in turn enhance their vocational identity in order to help college students achieve long-term sustainable occupational engagement in the uncertain social context.

## Data availability statement

The raw data supporting the conclusions of this article will be made available by the authors, without undue reservation.

## Ethics statement

The studies involving human participants were reviewed and approved by the Institutional Review Board, Normal College, Qingdao University. The patients/participants provided their written informed consent to participate in this study.

## Author contributions

ZG, XJ, and MZ designed, performed, analyzed the research, and wrote up the research. QQ and BS critically reviewed and edited the manuscript. All authors contributed to the article and approved the submitted version.
